# Right and Left Side-Lying Positioning During Bottle-Feeding in Premature Infants—A Randomized Crossover Pilot Study

**DOI:** 10.3390/jcm14145108

**Published:** 2025-07-18

**Authors:** Anna Raczyńska, Magdalena Suda-Całus, Tomasz Talar, Ewa Gulczyńska

**Affiliations:** Department of Neonatology, Neonatal Intensive Care Unit, and Neonatal Pathology, Polish Mother’s Memorial Hospital—Research Institute, 93-338 Łódź, Poland; madziasuda@wp.pl (M.S.-C.); tomtal@wp.pl (T.T.); ewagulcz@wp.pl (E.G.)

**Keywords:** premature infant, oral feeding, side-lying position, bottle-feeding

## Abstract

**Background/Objectives:** Optimal feeding position may contribute to improving the quality and safety of bottle-feeding in premature infants. The aim of this study was to compare the advantages of right side-lying (R-SLP) and left side-lying (L-SLP) positioning during the bottle-feeding of preterm infants. **Methods:** The randomized study included eight neonates (*n* = 8) born at ≤34 weeks of gestational age (GA). Four bottle-feeding sessions were conducted for each newborn: two in the R-SLP and two in the L-SLP position. Levels of oxygen saturation (SpO_2_) and heart rate (HR) were measured as indicators of physiological stability. The qualitative aspects of feeding included total time of SpO_2_ declines to ≤85%, the newborn’s alertness level based on the Neonatal Behavioral Assessment Scale (NBAS), and the number of possetings, regurgitations, and choking episodes. The volume of milk consumed and the duration of each feeding session were also recorded. **Results**: The L-SLP position was characterized with higher SpO_2_ (*p* = 0.042) at the 10th minute after feeding and lower HR (*p* = 0.022) at the end of feeding. Greater milk intake (*p* = 0.042), shorter feeding duration (*p* = 0.021), and shorter duration of SpO_2_ declines to ≤85% (*p* = 0.025) were also observed in L-SLP. No differences were found in alertness level, or in the number of choking episodes, possetings, or regurgitations compared to R-SLP. **Conclusions**: This pilot study suggests the potential efficacy of the L-SLP position during bottle-feeding of premature infants. The results require the need for larger studies to confirm the potential benefits of using L-SLP.

## 1. Introduction

Neurological and physiological immaturity in premature newborns contributes to difficulties in oral feeding. Short periods of activity, weak or delayed reflex reactions, impaired sucking–breathing–swallowing coordination, and difficulties in autoregulation are just some of the causes [1,2,3]. In most preterm infants, achieving full oral feeding is a stressful challenge for both the infant and caregivers [4,5,6]. Full, effective oral feeding is a prerequisite for discharge from the hospital [7,8]. High-quality feeding experiences in prematurely born infants may reduce stress in both infants and parents and help prevent long-term feeding disorders [9].

Determining the optimal position during bottle-feeding in prematurely born infants may positively influence the feeding process [10]. The search for an optimal bottle-feeding position for premature infants relates, on the one hand, to the feeding difficulties caused by multisystem immaturity and, on the other, to the frequent need for bottle-feeding during the transition from enteral to fully oral feeding. Side-lying position (SLP) is one of the approaches used in bottle-feeding-compared premature infants [11]. Previous research has indicated the safety and effectiveness of this position with the semi-elevated position (SEP), which is most commonly used in the bottle-feeding of newborns [12,13,14,15,16,17,18,19,20,21]. However, these pieces of research have limited evidential value due to small sample sizes, analysis of selected aspects [10,20], or slight differences in newborn positioning, which may affect the outcomes [16]. To date, only one study has compared right and left side-lying positions, focusing mainly on feeding efficiency [22]. In our opinion, comparing additional factors in both positions during bottle-feeding is necessary, as the side on which an infant is positioned during feeding may cause the occurrence of symptoms from the gastrointestinal system, as it has an asymmetric course in a human’s body.

In prematurely born infants, therapeutic positioning is used as developmental support. In incubators and beds, the position of a newborn is regularly changed to promote flexion, midline orientation, mouth–hand movement, and self-regulation [23], and to reduce stress levels [24]. This approach also helps prevent cranial distortions such as positional plagiocephaly and supports the development of body symmetry [25,26]. Feeding periods are especially important for muscle activation, which leads to correct movement patterns in favorable states of infant activity, as infants are wakeful and more active during feeding sessions. Positioning during bottle-feeding may also promote symmetrical stimulation and help avoid muscle-related asymmetry. Paying attention to body positioning in relation to infant body symmetry during feeding aligns with the assumptions of neurodevelopmental care and is one of the core goals of neuropediatric rehabilitation [27,28,29]. Because most caregivers use their dominant hand while bottle-feeding—and the majority are right-handed [30]—infants are typically fed in SLP on their left side, allowing the caregiver to hold the bottle in their right hand. This differs from breastfeeding, where infants are fed from both sides. Alternating the use of R-SLP and L-SLP promotes body symmetry. Therefore, applying both positions during bottle-feeding may support the proper development of immature newborns who are exclusively or predominantly bottle-fed for any reason.

Feeding by a right-handed person in the L-SLP position is more comfortable for the caregiver, as it is done with the dominant hand. Switching to the R-SLP and feeding with the left, non-dominant hand may increase muscle tension in the caregiver, related to uncertainty about the lower performance of the non-dominant hand [31] and exacerbated by parental stress associated with premature birth and the Neonatal Intensive Care Unit (NICU) environment [32]. It is well known that premature infants are more susceptible to external influences and stimuli [33]. Newborns may be sensitive to the quality of tactile stimuli from their environment due to their heightened tactile sensitivity [34]. Therefore, handedness is one factor worth considering when studying the effects of positioning on the right and left sides.

The purpose of the present study was to preliminarily assess the safety and effectiveness of both tested positions to validate their usefulness in the bottle-feeding process of prematurely born infants, as well as for neurorehabilitation and early intervention purposes.

## 2. Materials and Methods

This crossover experimental study, using an alternating randomized design (sealed envelope randomization), was conducted on eight (*n* = 8) premature infants born at ≤34 weeks of GA, who were hospitalized in a neonatal unit at a tertiary referral center between July 2021 and August 2021. These premature infants met all the inclusion criteria and did not meet any of the exclusion criteria established for the study.

Inclusion criteria were as follows: circulatory and respiratory stability; GA of ≤34 weeks; readiness for oral feeding; currently transitioning from enteral nutrition to full oral feeding; fed orally at least four times within 24 h; and use of one specific type of bottle and teat (50 mL Beldico bottle and Beldico pink ring teat for premature infants). The caregiver was required to be right-handed (i.e., with a dominant right hand).

Exclusion criteria were as follows: parental refusal to participate; bottle-feeding not being the parental preference; presence of congenital abnormalities or metabolic diseases; disorders likely to significantly affect feeding, such as cleft lip and/or palate, facial paralysis, or congenital defects of the facial skeleton; newborns after abdomen chirurgical treatment; low Apgar scores (<5 at the 5th and 10th minute after birth); administration of analgesics, anticonvulsants, or sedatives; <72 h since extubation prior to the trial; parenterally fed infants; infants with administered intravenous infusion with glucose.

One of the authors (A.R.) informed the parents about the purpose of the study and obtained their written informed consent. Enrolled infants were fed in two positions: R-SLP and L-SLP. Four bottle-feeding sessions were conducted for each newborn. The total number of study feeding sessions was thirty-two (*n* = 32). The position for the first feeding was randomly assigned and was changed after every session. Two consecutive feeding sessions were included in the study for each participant within a 24 h period to avoid excessive fatigue in the infants. There was a break of at least 24 h but less than 72 h between the first and second pair of feeding sessions to obtain maximally similar results while accounting for the increasing effectiveness of feeding associated with the maturation of feeding skills in premature infants.

**Figure 1:**
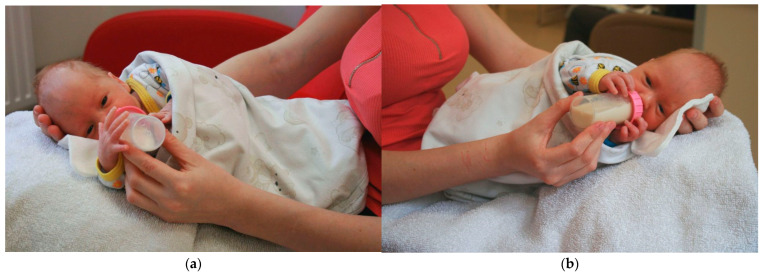
Photo of feeding positions used in the study. Photo (**a**): Right side-lying position (R-SLP). Photo (**b**): Left side-lying position (L-SLP).

Infants were fed on the caregiver’s legs in a slightly flexed posture and loosely wrapped. The infant’s head and neck were supported by the caregiver’s hand to maintain midline body symmetry. The angle between the horizontal plane and the infant’s midline was 30–45 degrees. Both positions are shown in Figure 1a,b.

The levels of SpO_2_ and HR were measured as indicators of the newborns’ physiological stability using a pulse oximeter (Masimo RAD97), with the sensor placed on the infant’s right foot. The factors determining the qualitative aspects of feeding included the total time of SpO_2_ declines to ≤85%; the newborn’s level of alertness according to the NBAS [35]; and the occurrence of choking episodes, possetings, and regurgitations. The proportion of milk consumed (volume of milk taken relative to the expected volume) and the duration of feeding and the entire feeding session were also analyzed. Feeding sessions were videorecorded.

The duration of the feeding session was defined as the total time of the study feeding intervention—that is, from the moment the infant was taken from the bed until they were returned to rest (in bed or on the parent’s chest)—including all pauses during feeding, such as breaks for burping or breathing equalization, if needed. The duration of feeding was calculated as the total time the teat was inserted intraorally during the session.

Choking episodes were defined as sucking–breathing–swallowing discoordination, characterized by coughing, interruption of feeding, and the possible presence of other feeding-related symptoms that may co-occur, such as desaturation, bradycardia, respiratory effort, and decreased activity of the newborn.

The level of activity was determined using the six-item NBAS scale. The less active the newborn, the lower the score on the scale, with successive points representing the following: 1—quiet sleep, 2—active sleep, 3—drowsy, 4—quiet alert, 5—active alert, and 6—crying [35].

The variables considered in the study were observed starting from 2 min before the beginning of feeding until 10 min after feeding ended. The following variables—SpO_2_, HR, and level of activity according to the NBAS scale—were measured at five time points: 2 min before feeding, in the 3rd minute of feeding, in the 10th minute of feeding, at the end of feeding, and 10 min after the end of feeding. The volume of food consumed was measured at the 10th minute and at the end of feeding. Choking episodes, regurgitation, possetings, and declines in SpO_2_ ≤ 85% were observed and recorded if they occurred at any point during the study.

The study was approved by the Bioethics Committee of the Polish Mother’s Hospital—Research Institute, Łódź, Poland (opinion number 46/2021). Written informed consent to participate in the clinical trial was obtained from the parents of all participating newborns. This study was registered on ClinicalTrials.gov ID: NCT04987983, registration date: 14 July 2021.

### Statistical Evaluation

The data obtained in the study were recorded in Microsoft Excel and subsequently analyzed using IBM SPSS v.26. Normality checks were conducted separately for the outcome measures. Histograms, boxplots, and Q–Q plots were visually assessed, along with the results of the Shapiro–Wilk test. The results suggested that most outcome variables were not normally distributed. Both parametric and nonparametric statistics were used in the analyses. Moreover, statistical corrections were applied to the parametric tests to account for any violations of their assumptions. The alpha level was set at α = 0.05.

## 3. Results

The mean GA at birth was 31.71 weeks (SD = 1.03), and at the start of the study, it was 35.39 weeks (SD = 0.89). All participants were delivered by cesarean section (CS). The study group included three male (37.5%) and five female (62.5%) newborns. The mean birth weight was 1570.00 g (SD = 286.36). None of the infants required respiratory support during the study, including non-invasive respiratory support or oxygen therapy. Detailed characteristics of the study group are presented in Table 1.

### 3.1. Oxygen Saturation

**Figure 2:**
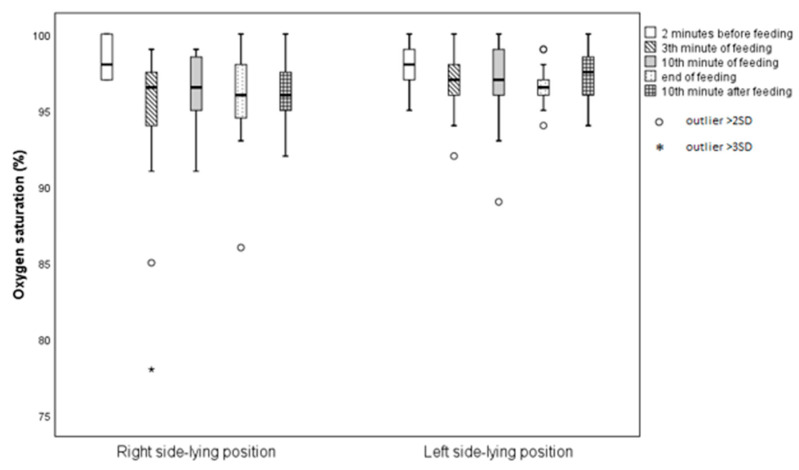
Mean levels of saturation with respect to infant’s position.

SpO_2_ fluctuations were compared between the R-SLP and L-SLP positions. A statistically significant difference was observed in oxygen saturation 10 min after feeding (*p* = 0.042). Infants fed in the L-SLP position had a higher SpO_2_ level (97.38% ± 1.89%) compared to those fed in the R-SLP position (96.31% ± 2.15%) (Figure 2).

### 3.2. Heart Rate

**Figure 3:**
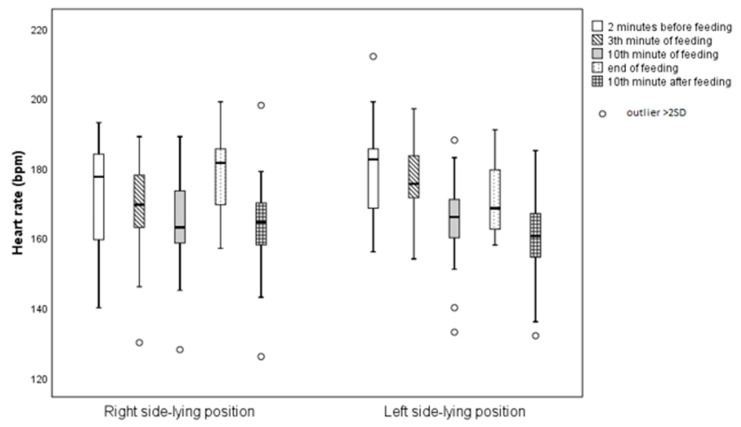
Average heart rate with respect to infant’s position.

The highest mean HR was observed in the L-SLP position 2 min before feeding (179.56 ± 14.86 bpm), while the lowest was recorded in the R-SLP position 10 min after feeding (163.63 ± 15.83 bpm). There was one statistically significant difference (*p* = 0.022) in HR between the L-SLP and R-SLP positions at the end of feeding. Infants fed in the R-SLP position had a higher HR (178.63 ± 12.57 bpm) compared to when fed in the L-SLP position (171.19 ± 9.79 bpm) (Figure 3).

### 3.3. Level of Activity

There were no statistically significant differences in the level of activity (according to NBAS). Nonetheless, the highest mean (4.56 ± 1.15) was observed in the L-SLP position 2 min before feeding, while the lowest (1.75 ± 0.58) was observed in the R-SLP position 10 min after feeding.

### 3.4. Proportion of Food Intake

**Figure 4:**
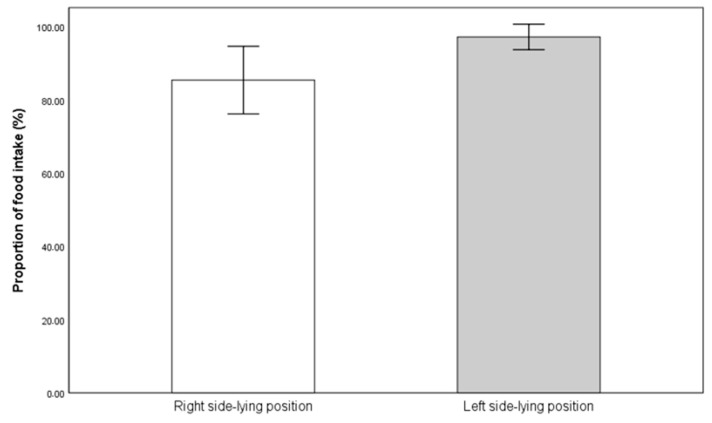
Mean proportion of milk intake in 10th minute of feeding.

**Figure 5:**
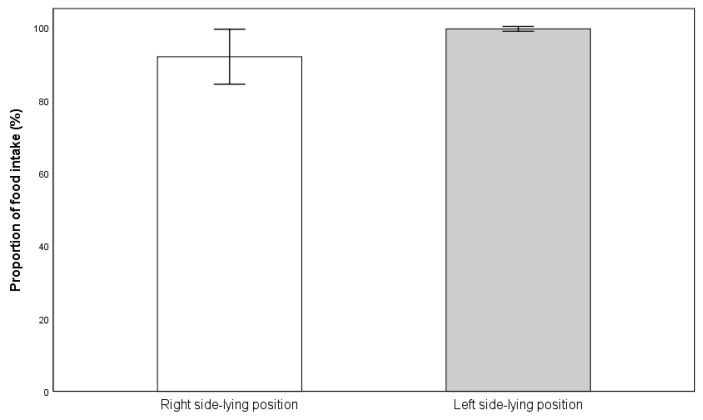
Mean proportion of milk intake at the end of feeding.

Next, the proportion of food intake was compared between the R-SLP and L-SLP positions. Infants fed in the L-SLP position at the 10th minute of feeding (97.26% ± 6.55%) consumed statistically significantly more than when fed in the R-SLP position (85.47% ± 17.34%) (*p* = 0.013) (Figure 4). There was also a statistically significant difference in the proportion of food intake at the end of feeding. Infants fed in the L-SLP position (99.69% ± 1.25%) consumed significantly more compared to those fed in the R-SLP position (92.02% ± 14.18%) (*p* = 0.042) (Figure 5).

### 3.5. Time of the Feeding

**Figure 6:**
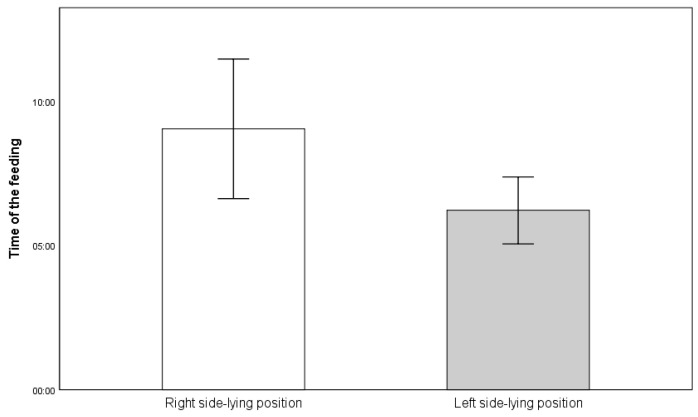
Mean time of feeding (in minutes:seconds).

Feeding time between the R-SLP and L-SLP positions was compared. Infants fed in the R-SLP position (9 min 2 s ± 4 min 31 s) were fed statistically significantly longer (*p* = 0.024) compared to those fed in the L-SLP position (6 min 13 s ± 2 min 10 s) (Figure 6).

### 3.6. Time of the Feeding Session

**Figure 7:**
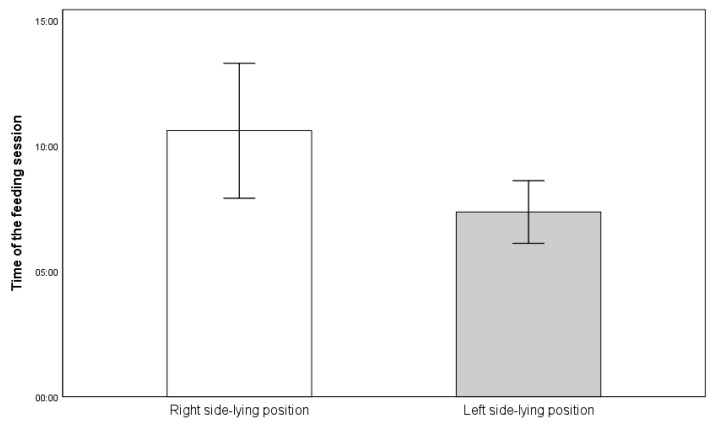
Mean time of feeding session (in minutes:seconds).

The duration of the feeding sessions was also compared. A statistically significant difference was found (*p* = 0.021). Infants fed in the R-SLP position (10 min 37 s ± 5 min 3 s) had a longer mean feeding session duration compared to when fed in the L-SLP position (7 min 22 s ± 2 min 20 s) (Figure 7).

### 3.7. Time of the Saturation Decrease to the Level of ≤85%

**Figure 8:**
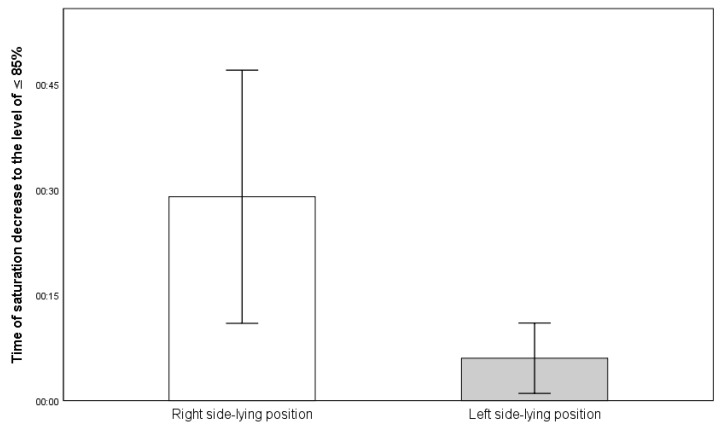
Mean time of saturation declines to ≤85% (in minutes:seconds).

Infants fed in the R-SLP position (29 ± 34 s) had a significantly longer mean duration of SpO_2_ decline to ≤85% compared to those fed in the L-SLP position (6 ± 9 s) (*p* = 0.025) (Figure 8). 

### 3.8. Feeding Intolerance

Based on the results of chi-squared tests, the R-SLP or L-SLP positions were not significantly associated with the number of choking episodes (*p* = 0.202), possetings (*p* = 0.333), or regurgitations (*p* = 0.083).

## 4. Discussion

The comparison of SLP with the more commonly used SEP or similar supine positions—such as cradled, semi-upright, or semi-elevated upright positions—for feeding prematurely born newborns has been the subject of several previous studies [12,13,14,15,16,17,18,19,20,21]. It should be noted that not all studies included photographic documentation of the positions used, and those that did showed variations in newborn positioning in both SLP and SEP (or their modifications), which may have influenced the results. Although reports indicate that SLP is safe and may benefit prematurely born infants, there is still no clear evidence confirming its advantages, despite its use and occasional recommendation as an optimal feeding position for preterm infants [36,37].

A comparison of SLP on the right and left sides has so far been conducted by only one research team. That study did not show significant differences between left (L-ESL) and right (R-ESL) semi-elevated positions in terms of food volume consumed, feeding duration, feeding efficiency, or mean scores on the Early Feeding Skills Assessment—Turkish version (EFS-Turkish) [22]. However, that study did not assess several parameters that appear important when comparing both positions. Considering physiological indicators (SpO_2_, HR) and feeding intolerance factors is particularly relevant in premature infants. According to the description in the cited study (which did not include photographs), the only difference compared to our study was the higher positioning of newborns: the angle of the body in relation to the horizontal plane was 45–60° in both L-ESL and R-ESL positions, while it was 30–45° in both L-SLP and R-SLP in our study [22]. In our study, a greater volume of food was consumed in the L-SLP position at both the 10th minute of feeding and at the end of feeding. Consequently, the feeding and overall session durations were also shorter in the L-SLP. The shorter duration of the feeding session in the L-SLP position was almost entirely due to the reduced feeding time compared to the R-SLP position. However, this conclusion contradicts the results of the previously cited study [22], which may be due to the higher infant positioning used in that research. It is possible that the steeper angle (45–60° versus 30–45°) may increase abdominal cavity compression due to gravity. This hypothesis should be further investigated in future clinical studies.

In our study, the higher mean SpO_2_ level at the 10th minute after feeding and the higher mean HR at the end of feeding in the R-SLP position may indicate that infants fed in the L-SLP position were less fatigued during and after feeding. This may be related to the shorter feeding time observed in the L-SLP position. Additionally, the total duration of SpO_2_ declines to ≤85% was significantly lower in L-SLP. For these reasons, feeding in the L-SLP position appears to be more comfortable for bottle-fed newborns, potentially due to reduced fatigue associated with the shorter duration of both feeding and the overall session.

In our study, the NBAS scale [35] was used to determine the activity state in both positions and did not reveal any statistically significant differences. Monitoring the newborn’s activity state during feeding is important for improving the care of premature infants hospitalized in the NICU [15,16,20,38].

A slight advantage of L-SLP in terms of caregiver handedness suggests a need for future research involving a control group of left-handed caregivers, to help rule out factors that may influence the results. In our opinion, the handedness of the caregivers may be important [30,31] because it cannot be ruled out that the greater comfort of the caregiver when feeding the baby with the right, dominant hand in the L-SLP may influence the feeding process.

Previous studies have investigated the influence of positioning on the right or left side of the body after feeding, particularly in the context of gastroesophageal reflux (GER) or suspected gastroesophageal reflux disease (GERD) [39], as well as in the assessment of gastric residuals during the feeding process in premature infants [40]. Although these studies, along with our own research hypothesis, relate to gastrointestinal outcomes, positioning in either R-SLP or L-SLP during bottle-feeding did not result in significant differences in symptoms potentially related to GER/GERD—such as regurgitations, possetings, or choking episodes—in our current study. However, the longer duration of oxygen saturation declines to ≤85% observed in the R-SLP position, along with the higher HR at the end of feeding, could be considered potential indicators of GER/GERD exacerbated by R-SLP positioning [39,40,41]. A major limitation of our study, however, was the use of the least invasive yet also less objective method—observational assessment of these adverse events.

Previous studies comparing SLP with the SEP position have shown a slight advantage of SLP in terms of physiological stability: SLP promotes higher SpO_2_ level and decreases variability in HR level [10,14,17,19]. SLP also decreases the work of breathing, minimizing premature infant’s stress and promotes suck–swallow–breathe coordination [10,21]. Infants fed in the SLP position also consumed greater volumes of milk and experienced fewer choking episodes compared to those fed in the SEP position [15]. Studies that examine the occurrence of choking episodes, penetrations, or aspirations in SLP versus SEP are particularly valuable for this vulnerable population [15,16,42,43]. Choking episodes are among the most stressful events during feeding, for both premature infants and their caregivers [15]. These episodes may also indicate penetration or aspiration into the airway [21,42] and can lead to complications such as aspiration pneumonia [15]. Therefore, identifying a feeding position that reduces the frequency of such adverse events is crucial for improving care quality and ensuring feeding safety. It can also support positive and high-quality feeding experiences by reducing the risk of airway aspiration [4,21].

The most valuable study to date on the use of the SLP in newborns is the preliminary assessment of its potential as a therapeutic position for premature infants with dysphagia [42]. This study used modified barium swallow (MBS) and radiologic imaging of the oropharynx during bottle-feeding to evaluate the act of swallowing. In six out of nine newborns, the SLP significantly improved airway protection; in the remaining three, no difference was found between the semi-elevated SLP (with a 30-degree angle to the horizontal plane) and the reclined-upright position [42]. The researchers did not specify which side the infants were positioned on during SLP, but the accompanying photo suggests they were placed on the left side. This is a promising research direction, though limited by the patient profile, as ionizing radiation precludes the inclusion of healthy newborns as a control group [42,43].

Another study, using fiberoptic endoscopic evaluation of swallowing (FEES), was conducted in breastfeeding newborns with laryngomalacia to evaluate the impact of positioning on feeding [44]. This research compared two positions during breastfeeding, finding that the semi-prone position reduced the risk of aspiration compared to supine or semi-lateral decubitus positions. Although the study differs in feeding method (breastfeeding vs. bottle-feeding) and position tested, it confirms that body positioning may influence the feeding process [12,13,14,15,16,17,18,19,20,21,44]. FEES, while less invasive than MBS due to the absence of radiation exposure, still cannot be used routinely and is reserved for cases requiring in-depth diagnostics [45]. Previous studies initially indicate that the greatest benefits from the use of the SLP are obtained by newborns with co-occurring difficulties in feeding (episodes of choking, dysphagia), and it seems that, in selected clinical situations, the use of the SLP can have a significant impact on improving the quality and safety of feeding [15,16,43], which may indicate the direction of future research in the area of searching advantages for SLP applications.

The similarity between the SLP and positions used during breastfeeding has been identified as an advantage of this strategy when applied to bottle-feeding [15,17]. Breastfed newborns are typically positioned in SLP on both the right and left sides. However, important differences exist between breastfeeding and bottle-feeding, including breathing patterns and milk flow rate [46,47,48]. Evaluating the use of SLP on the right or left side during bottle-feeding may be particularly relevant for newborns who are not breastfed and could benefit from consistent or side-specific positioning. This may be important in specific clinical situations in bottle-feeding infants, for example, after thoracic or abdominal surgery in infants located on one side of the newborns body, or in cases of asymmetry of the infants body or braincase. An attempt to use SLP for early intervention as a pediatric physiotherapy support is worth considering.

Research on the impact of positioning, taking into account the SLP, was also carried out in adults. Using two-dimensional echocardiography, Doppler, tissue Doppler imaging, and arterial pressures using a volume-clamp method, the cardiac performance was enhanced in the left lateral position versus the supine position [49]. Another piece of research was conducted in adult patients with dilated cardiomyopathy [50]. Patients with heart failure during sleep prefer lying on their right side and complain of increasing dyspnea when adopting the left lateral position, which is explained by hemodynamic changes. This study has shown that body position influence on the echocardiographic left ventricular outflow tract time-velocity integral (LVOT-TVI) and tricuspid annular plane systolic excursion (TAPSE) values are functional parameters of left and right ventricular function [50]. Despite the significant differences between premature newborns and adult patients, it may be necessary to take into account cardiac factors as aspects that may influence the course of feeding in the L-SLP and R-SLP.

This pilot study suggests the potential efficacy of the L-SLP position during the bottle-feeding of premature infants. The results require the need for larger studies to confirm the potential benefits of using L-SLP.

The authors emphasize that all mothers of infants enrolled in the present study were informed of the health benefits of breastfeeding [48,51] and were encouraged to breastfeed. Skin-to-skin contact was also promoted, as it is a well-established practice in NICUs with multiple benefits for both parents and premature infants [51,52,53].

## 5. Limitations

There are several limitations of this pilot study. A major limitation is the small sample size. The results of statistical analyses based on a small sample are inherently less reliable and less objective than those based on a larger sample. Due to the limited sample size, the infants could not be grouped according to birthweight, birth age, or other characteristics. Future studies should include a larger sample size to enable additional analyses. It may also be worth considering an expansion of the exclusion criteria to omit, for example, newborns who have recently had an infection, received antibiotic therapy, or have certain neurological backgrounds (such as grade III–IV IVH or periventricular leukomalacia—PVL) that would warrant exclusion. In our opinion, newborns in good clinical condition with grade I or II IVH could be included in the study, but this should be clarified. If necessary, outcomes for this subgroup should be analyzed separately and compared with those of newborns without imaging abnormalities in the central nervous system. We also did not identify birthweight as one of the inclusion criteria by establishing, for example, a specific percentile range. Including such criteria could improve the homogeneity of the study group. Another limitation was the lack of defined criteria for resuming the study after a break for breathing stabilization. It is necessary to specify when the study should be continued (e.g., upon reaching a particular heart rate or oxygen saturation level), especially since the newborns were monitored during the study via pulse oximetry. This would help decide to resume feeding after a break more objectively. While several studies have examined feeding in the SLP, only one previous study has compared right and left lateral feeding, and it evaluated only a limited number of factors. This restricted our ability to contextualize our findings. Further research with a larger sample size is therefore warranted.

## 6. Conclusions

This pilot study suggests the potential efficacy of the L-SLP position during the bottle-feeding of premature infants. According to the findings of this study, infants fed in the L-SLP position were characterized by higher SpO_2_ levels at the 10th min after feeding and lower heart rates at the end of feeding. Greater milk intake, shorter feeding duration, and shorter durations of SpO_2_ declines to ≤85% were also observed in L-SLP. No differences were found in alertness level or the number of choking episodes, possetings, or regurgitations compared to R-SLP. Because this is a pilot study with a small sample size, its results should be interpreted with caution. Nonetheless, the results suggest a need for larger studies to further examine the potential benefits of using L-SLP in improving feeding performance.

## Figures and Tables

**Table 1:** Participants characteristics.

Participant	Delivery	Sex	Birth Weight	GA at Birth	PMA on First Tested Feeding	Neurological Background	Respiratory Support During Study
1	CS	F	1650 g	32	36 + 3/7	None	None
2	CS	M	1300 g	31	35 + 2/7	None	None
3	CS	F	1670 g	32	36 + 3/7	None	None
4	CS	M	1500 g	31	35 + 2/7	None	None
5	CS	F	2170 g	31 + 5/7	33 + 4/7	None	None
6	CS	F	1600 g	33	35 + 3/7	None	None
7	CS	F	1270 g	33	35 + 4/7	IVH I	None
8	CS	M	1400 g	30	35 + 1/7	None	None

Abbreviations used in a table: CS—caesarian section; F—female; M—male; g—grams; GA—gestational age; PMA—postmenstrual age; IVH—intraventricular hemorrhage.

## Data Availability

Data available after reasonable request.
